# Land use land cover change in the African Great Lakes Region: a spatial–temporal analysis and future predictions

**DOI:** 10.1007/s10661-024-12986-4

**Published:** 2024-08-27

**Authors:** Naomie M. Kayitesi, Alphonce C. Guzha, Marj Tonini, Gregoire Mariethoz

**Affiliations:** 1 Institute of Earth Surface Dynamics, Faculty of Geosciences and Environment, University of Lausanne, 1015 Lausanne, Switzerland; 2 International Union for Conservation of Nature (IUCN), East and Southern Africa Region, KN 16 Ave, Kigali, Rwanda

**Keywords:** Lake Kivu catchment; Explanatory variables; Seasonal composites; Future scenarios, Machin learning, Green growth economy

## Abstract

The African Great Lakes Region has experienced substantial land use land cover change (LULCC) over the last decades, driven by a complex interplay of various factors. However, a comprehensive analysis exploring the relationships between LULCC, and its explanatory variables remains unexplored. This study focused on the Lake Kivu catchment in Rwanda, analysing LULCC from 1990 to 2020, identifying major variables, and predicting future LULC scenarios under different development trajectories. Image classification was conducted in Google Earth Engine using random forest classifier, by incorporating seasonal composites Landsat images, spectral indices, and topographic features, to enhance discrimination and capture seasonal variations. The results demonstrated an overall accuracy exceeding 83%. Historical analysis revealed significant changes, including forest loss (26.6 to 18.7%) and agricultural land expansion (27.7 to 43%) in the 1990–2000 decade, attributed to political conflicts and population movements. Forest recovery (24.8% by 2020) was observed in subsequent decades, driven by Rwanda’s sustainable development initiatives. A Multi-Layer Perceptron neural network from Land Change Modeler predicted distinct 2030 and 2050 LULC scenarios based on natural, socio-economic variables, and historical transitions. Analysis of explanatory variables highlighted the significant role of proximity to urban centers, population density, and terrain in LULCC. Predictions indicate distinct trajectories influenced by demographic and socio-economic trends. The study recommends adopting the Green Growth Economy scenario aligned with ongoing conservation measures. The findings contribute to identifying opportunities for land restoration and conservation efforts, promoting the preservation of Lake Kivu catchment’s ecological integrity, in alignment with national and global goals.

**Graphical Abstract:**

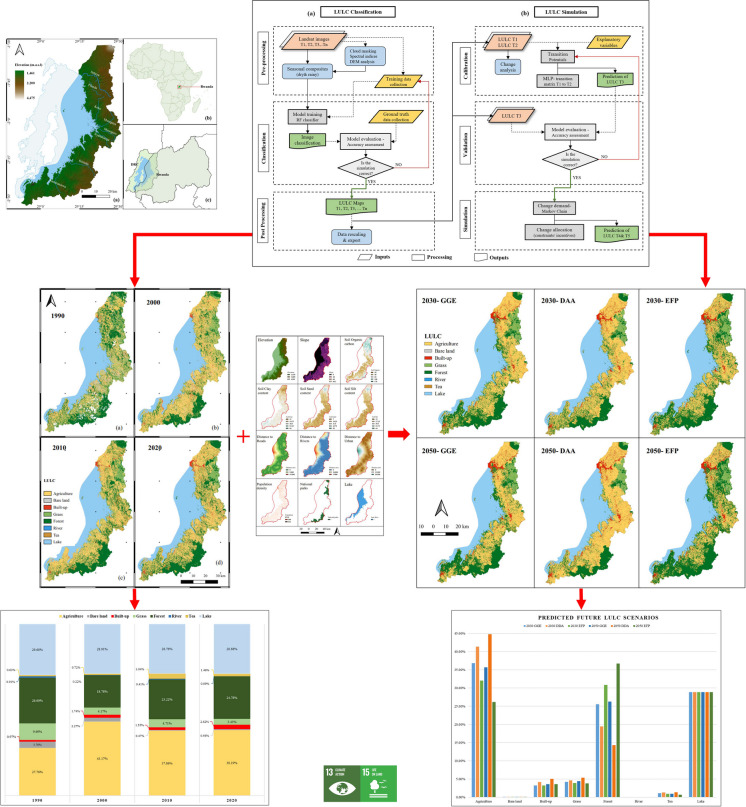

**Supplementary Information:**

The online version contains supplementary material available at 10.1007/s10661-024-12986-4.

## Introduction

Rwanda has initiated various environmental measures to restore the landscape’s ecological functionality, in recognition of the value of landscapes and ecosystems to national development. The Country became an early adopter of the global Bonn Challenge Initiative in 2011, committing to restore 2 million hectares of land, in addition to the Paris Agreement, Sustainable Development Goals, and Africa Agenda 2063 commitments (Dave et al., 2018). These efforts align with Rwanda’s Vision 2020 and 2050, the Economic Development and Poverty Reduction Strategy, and the Green Growth and Climate Resilience Strategy, to enhance the environmental sustainability in the country’s development (Banerjee et al., 2020). The 2019 forest cover mapping report indicates that about 30.4% of the country’s land is forested, an increase from 25% in 2009 (MoE, 2019). These statistics indicate progress in enhancing environmental sustainability within the country’s development agenda.

However, landscapes and ecosystems continue to face major threats primarily due to land use land cover change (LULCC) and exacerbated by climate change. LULCC, a process that alters the earth’s surface, is a pressing global environmental issue. Since the post-1950 era, anthropogenic activities are transforming the earth’s ecosystem structure and processes at a “great acceleration” (Steffen et al., 2015). The African Great Lakes Region is also undergoing these changes, facing substantial impacts due to human activities (Cohen et al., 2019). The main contributors to LULCC in this region are a complex interplay of political, demographic, and socio-economic factors arising, in part, from post-colonial actions. In particular, the Lake Kivu (LKV) catchment, located between Rwanda and Democratic Republic of Congo, has undergone extensive LULCC in recent decades. This is partly attributed to the conflicts and civil wars that happened in the region in the 1990s (Bagalwa et al., 2016; Plumptre, 2003). These changes contributed significantly to landscape’s destruction and changes, mainly deforestation, land degradation, and uncontrolled development activities. Rwanda, as a developing country with limited land resources and high population density, has recently experienced rapid population growth and economic development, leading to further significant landscape transformations (Li et al., 2021; Mugiraneza et al., 2019).

The LKV catchment occupies the country’s highest and wettest mountainous area, with steep topography (Kayitesi et al., 2022). The expansion of settlements, pastureland, and farmlands in forested areas, combined with heavy rainfall, has made this catchment prone to several environmental disasters (Karamage et al., 2017; Muhire et al., 2021; Uwihirwe et al., 2020). For example, the catchment experiences the highest level of landslides in the country (Nsengiyumva et al., 2018), and is highly susceptible to flooding (Bizimana & Sönmez, 2015). Additionally, it is at high risk of soil erosion, sediment transport, mass movement, and formation of gullies (Rukundo et al., 2018). According to the IUCN (2022) report, Rwanda experiences an annual loss of about 27 million tons of fertile soil, primarily affecting the districts in the western province. Notably, the LKV catchment is estimated to undergo a soil loss of approximately 116 t/ha/year (GIZ, 2020). Several studies in the literature have found a correlation between environmental disasters and LULCC (Foley et al., 2005; Guzha et al., 2018; Kayitesi et al., 2022; Mariye et al., 2022).

Understanding and identifying historical and future LULC dynamics over time, especially in rapidly evolving landscapes influenced by anthropogenic and natural factors, plays an important role in land development decision-making and planning (Selmy et al., 2023). Previous LULCC studies in Rwanda have predominantly utilized national data from the Regional Centre for Mapping of Resources for Development (RCMRD) to understand the landscape changes in the last decades (Arakwiye et al., 2021; Bagstad et al., 2020; Kulimushi et al., 2021; Li et al., 2021).

The lack of reliable or comprehensive data, along with the low resolution and quality of national datasets, underscores the need for improved and updated data to analyse LULC dynamics across different landscapes (Nedd et al., 2021). Namely, while the RCMRD dataset provides useful insights into LULCC in the catchment, finer-resolution studies are necessary to capture recent changes, including ongoing landscape restoration measures. These studies are required to provide detailed information for local-scale implementation, such as landscape rehabilitation and restoration measures. In addition, a deep investigation of the complex relationships linking LULCC and explanatory variables in the region remains unexplored, necessitating a comprehensive analysis. Comprehensive information on LULC transitions and their causes is important for regulatory purposes and the design of appropriate land management approaches (Mariye et al., 2022). However, in Rwanda, as in other parts of the world, basic data on LULC trends is still scarce, as a result, the consequences of this ongoing process are not clearly understood.

Research has shown that integrating remote sensing, Geographic Information System, and simulation modelling, offers a cost-effective approach for both qualitative and quantitative analysis of historical LULC patterns and the simulation of potential future LULC scenarios (Faruque et al., 2022; Mariye et al., 2022; Shafie et al., 2023). Cloud computing platforms like Google Earth Engine, which provide access to a wide range of remote sensing data, offer high computation power for time-series analysis (Adepoju & Adelabu, 2020; Nasiri et al., 2022). Various machine learning classification algorithms have been increasingly developed and widely used for image classification, with random forest identified as achieving higher mapping accuracy (Mellor et al., 2013; Sibanda & Ahmed, 2021; Talukdar et al., 2020). Additionally, specific band combinations and spectral indices have been recognized for enhancing feature identification in heterogenous landscapes (Ibrahim, 2023; Onyango & Opiyo, 2022). Moreover, several models and software packages have been developed to assess the relationship between historical LULCC and their explanatory variables, enabling the prediction of potential future scenarios (Gaur et al., 2020; Li et al., 2020). Among these, the Land Change Modeler (LCM), leveraging Multi-Layer Perceptron (MLP) artificial neural network, stands out as a robust tool for change analysis and predict future LULC scenarios by incorporating explanatory variables (Bakr et al., 2022; Hasan et al., 2020; Shafie et al., 2023; Sibanda & Ahmed, 2021).

This study aims to bridge the research gaps by (i) using remote sensing technology coupled with predictive learning to reconstruct historical LULC from 1990 to 2020 and analyse changes over time; (ii) examining the explanatory variables underlying landscape changes; and (iii) predicting potential future LULC scenarios for 2030 and 2050, based on three different development pathways. Namely, in the LKV catchment, various forms of LULCC are inevitable, due to the high population increase and socio-economic developments. In this context, the present study proposes a robust methodology for the detailed assessment of LULCC at the local scale offering valuable insights to support the planning of future land use, to adapt to anticipated environmental and socio-economic changes, aligned with national and international development goals and strategies.

## Materials and methods

### Study area

**Fig. 1:**
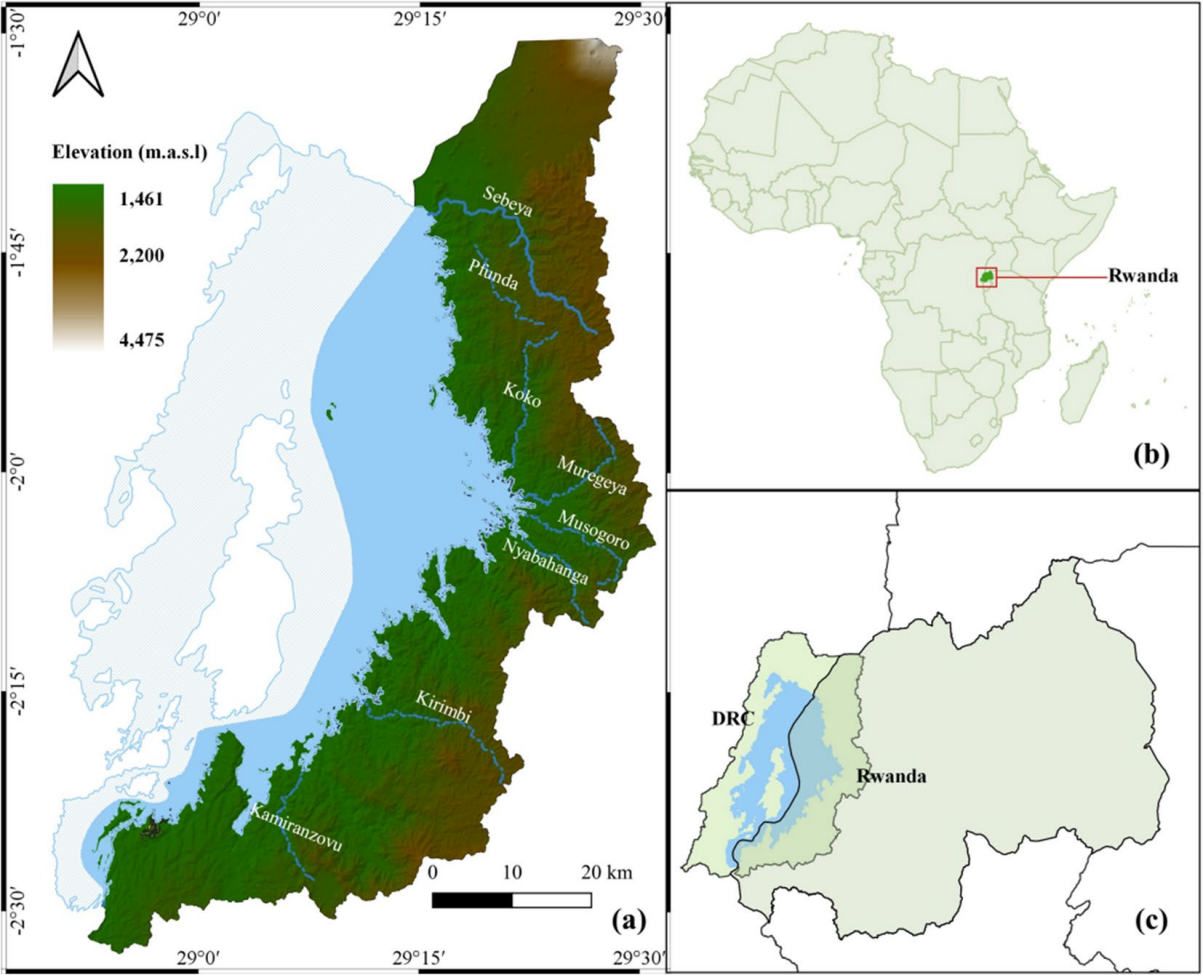
Study area: **a** Lake Kivu catchment on the Rwandan side; **b** the geographical location of Rwanda; and **c** the drainage basin of Lake Kivu spanning both Rwanda and Democratic Republic of Congo (GCS: WGS 84)

Lake Kivu is one of the African Great Lakes, located in the western branch of the East African Rift within the Albertine Rift region. The lake, covering an area of 2370 km^2^, has a watershed drainage area of 4940 km^2^. This is a transboundary region of Rwanda and Democratic Republic of Congo (Fig. 1c), part of the larger Congo basin. In Rwanda, the LKV catchment lies on the western part of the Congo-Nile Divide, spanning latitudes 1°30′S and 2°32′S, and longitudes 28°52′E and 29°31′E. The catchment covers 2425 km^2^ in Rwanda, including the lake surface.

The LKV catchment is characterized by mountainous and hilly terrain, interrupted by river valleys. Elevation in the catchment rises from 1461 at the lake shore, to 4475 m.a.s.l. in the northern mountains (Fig. 1a). The topography exhibits significant variations in slope (ranging from 0.13 to 86.3%), with most slopes exceeding 40%. The catchment experiences a humid tropical climate with average temperatures ranging from 16 to 20 °C, with little seasonal variation. The area is renowned for high precipitation and low temperatures, making it the wettest and coldest region in Rwanda. Rainfall is characterized by both spatial and temporal irregularities, with the driest part of the catchment receiving around 1200 mm of annual rainfall, while the wettest areas, particularly the natural forest parks, receive as much as 2300 mm (Balagizi et al., 2022). The rainfall follows a bimodal pattern, with two distinct rainy seasons from March to May and from September to December, alternating with two dry seasons.

Rwanda’s soils, resulting from physico-chemical alteration of granite, gneiss, quartz, schist, and volcanic rocks, are naturally fragile (Nambajimana et al., 2019). According to FAO soil classification, the dominant soil types in the LKV catchment are Acrisols with scattered occurrences of Cambisols. Andosols are found in the northern region, which are relatively fertile and suitable for intensive farming, but are also susceptible to erosion (Mwanjalolo et al., 2015). Luvisols are predominant in the central part of the catchment. These fertile soils and the high annual rainfall make this region a prominent agricultural area, with major crops being maize, wheat, beans, irish potatoes, and sweet potatoes (Akinyemi, 2017; Ekise et al., 2013). The LKV catchment is renowned for its rich biodiversity, encompassing three of Rwanda’s four national parks. Gishwati-Mukura National Park is known for its diverse ecosystems including several endangered species. Nyungwe National Park, in the south, is one of Africa’s largest protected mountain rainforests, sheltering a wide range of plants and animals. The Volcanoes National Park in the north is known for its population of critically endangered mountain gorillas, as well as other wildlife species (Kanyamibwa, 1998).

### Input data

Satellite images analysed in the present study come from Landsat imagery, chosen because of their long operational periods, the large spectral sensitivity, and good resolution (30 by 30 m). Images cover the period of 1988–2020 with time span for the acquisition each 10 years (Fig. 3). The Digital Elevation Model (DEM), with a resolution of 90 by 90 m, was provided by the (RCMRD, n.d.). Other geospatial datasets, namely the road networks, the river networks, and national parks boundaries, were acquired from the Africa Geoportal hosted by the Rwanda Land Management and use Authority (RLMA, n.d.). Population density information with 100 by 100 m grid cell, was downloaded from (WorldPop, n.d.). Soil data, such as soil texture layers (clay, sand, and silt content) and organic carbon, were acquired from the Africa Soil Information Service at 250 m resolution (ISRIC, n.d.). To ensure a consistent geospatial reference and seamless integration, all the digital geographical layers were geo-referenced to UTM Zone 35S and rescaled to a uniform resolution of 30 by 30 m, establishing a harmonized geospatial framework for the analysis.

### Methods

**Fig. 2:**
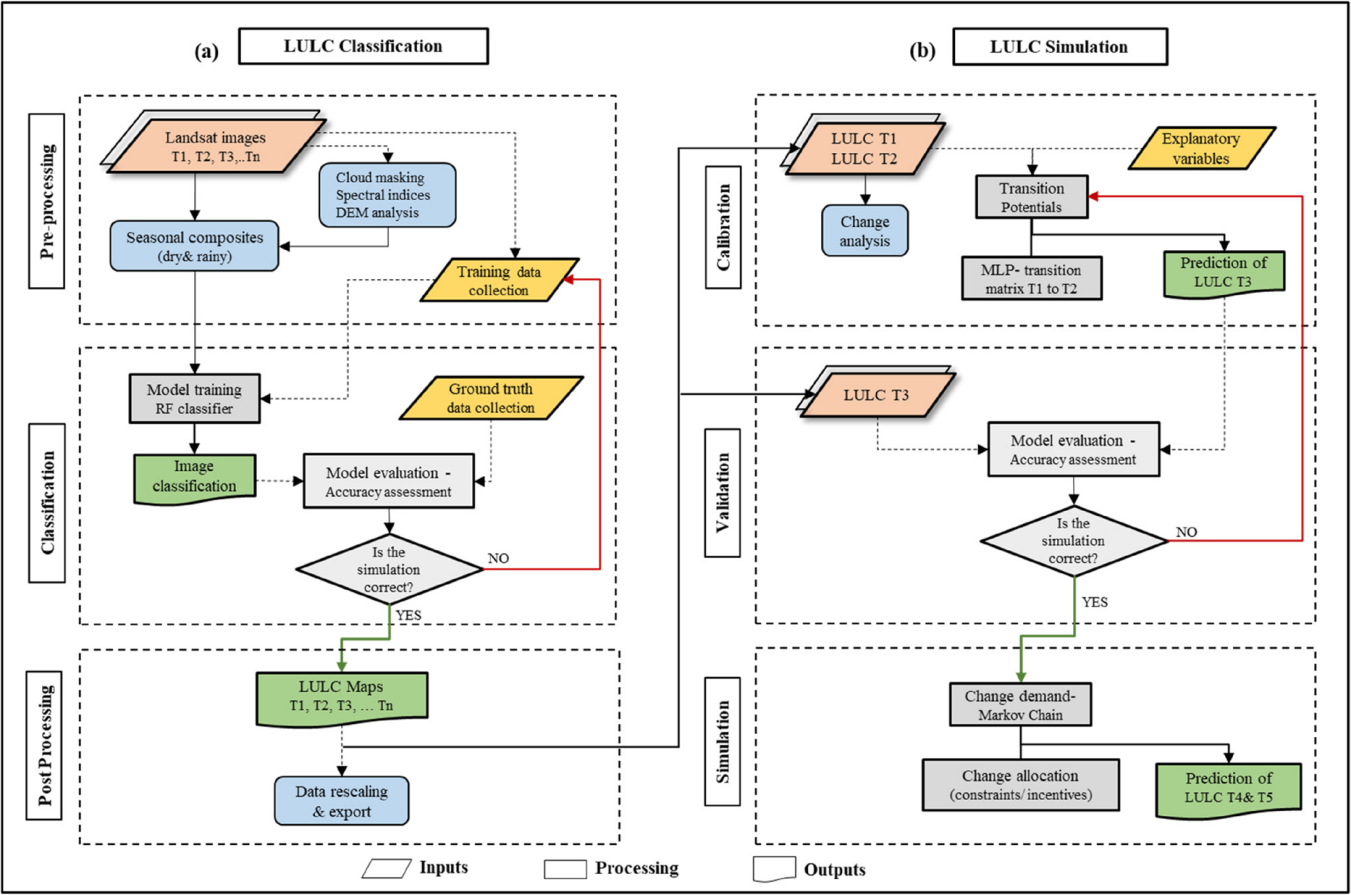
Methodological workflow applied for **a** LULC classification, and **b** prediction of future LULC scenarios

The methodology implemented in the present study includes two main steps: the first part encompasses the image classification carried out in Google Earth Engine (Fig. 2a) performed using random forest, an ensemble learning method. The second part involves predicting future LULC scenarios achieved by combining the observed LULC class transitions with explanatory variables into a predictive learning system based on MLP (Fig. 2b).

### Image classification and change detection

**Fig. 3:**
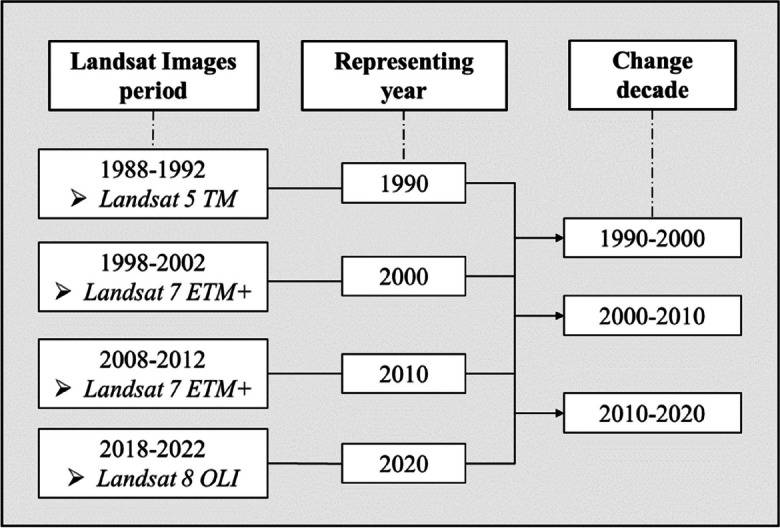
Timeline of image classification periods with corresponding Landsat sensors

A supervised classification approach was adopted, utilizing random forest classifier. The process was conducted using the Code Editor of Google Earth Engine, a cloud-based platform that provides access to a wide range of remote sensing datasets. Due to the limited availability and cloud cover of images in 1990, the LULC for 1990 was derived from images taken between 1988 and 1992. To maintain consistency, the same approach was applied to subsequent periods, each corresponding to different Landsat sensors. Change detection was then performed in three decades. Figure 3 illustrates the Landsat images used during each period, the corresponding years they represent, and the change decades they encompass.

#### Image pre-processing

The accuracy of supervised classification in remote sensing heavily relies on factors like the quality of training, image resolution, and the choice of classifier (Chen & Stow, 2002; Foody & Mathur, 2004). However, satellite images are often subjected to distortion caused by sensor, solar, atmospheric, and topographic effects (Young et al., 2017). While some studies suggest using surface reflectance to account for atmospheric effects, top of atmosphere reflectance has proven more consistent in mountainous regions (Chen & Zhu, 2022; Flood, 2014). Therefore, this study utilized calibrated top of atmosphere reflectance from collection 1 Tier-1 Landsat scenes, which are known for their high-quality data and suitability for time-series analysis.

It is crucial to use cloud-free images to ensure reliable results. However, given that the study region is located in mountainous region near the equator, cloud cover is a persistent challenge, making the classification process more difficult (Zaidi et al., 2017). To mitigate this issue, the cloud score algorithm was employed to selectively filter images, retaining only those with a cloud cover of less than 20%. This threshold was chosen to balance the preservation of data integrity with the need of minimizing the influence of clouds on the analysis. This specific threshold value was determined through experimentation, considering the trade-offs between data retention and cloud removal. In tropical regions, especially for data from earlier years like the 1980s and 1990s, acquiring cloud-free images presents a significant challenge (Akinyemi, 2017). Consequently, for the initial study period, the permissible cloud cover threshold was raised to 30% to accommodate these constraints.

To enhance landscape feature identification based on unique spectral characteristics, three spectral indices were added to the Landsat bands. These are (1) Normalized Difference Vegetation Index (NDVI), used to detect the presence of vegetation and evaluate its health by comparing the red band (absorption of chlorophyll), and near infrared band (reflectance of the vegetation canopy); (2) Normalized Difference Water Index (NDWI), applied to differentiate water from dry land, based on difference in reflectance between the near infrared and green bands; and (3) Normal Difference Build-up Index (NDBI), designed to highlight the presence of built-up areas, that reflects more short-wave infrared than in the near infrared (Faruque et al., 2022; Lee et al., 2018; Onyango & Opiyo, 2022; Triscowati et al., 2019). Additionally, slope and elevation bands were incorporated to further distinguish various LULC patterns. These features have been identified as crucial for LULC classification, offering insights into terrain features and associated landscape patterns (Adepoju & Adelabu, 2020; Ibrahim, 2023).

**Fig. 4:**
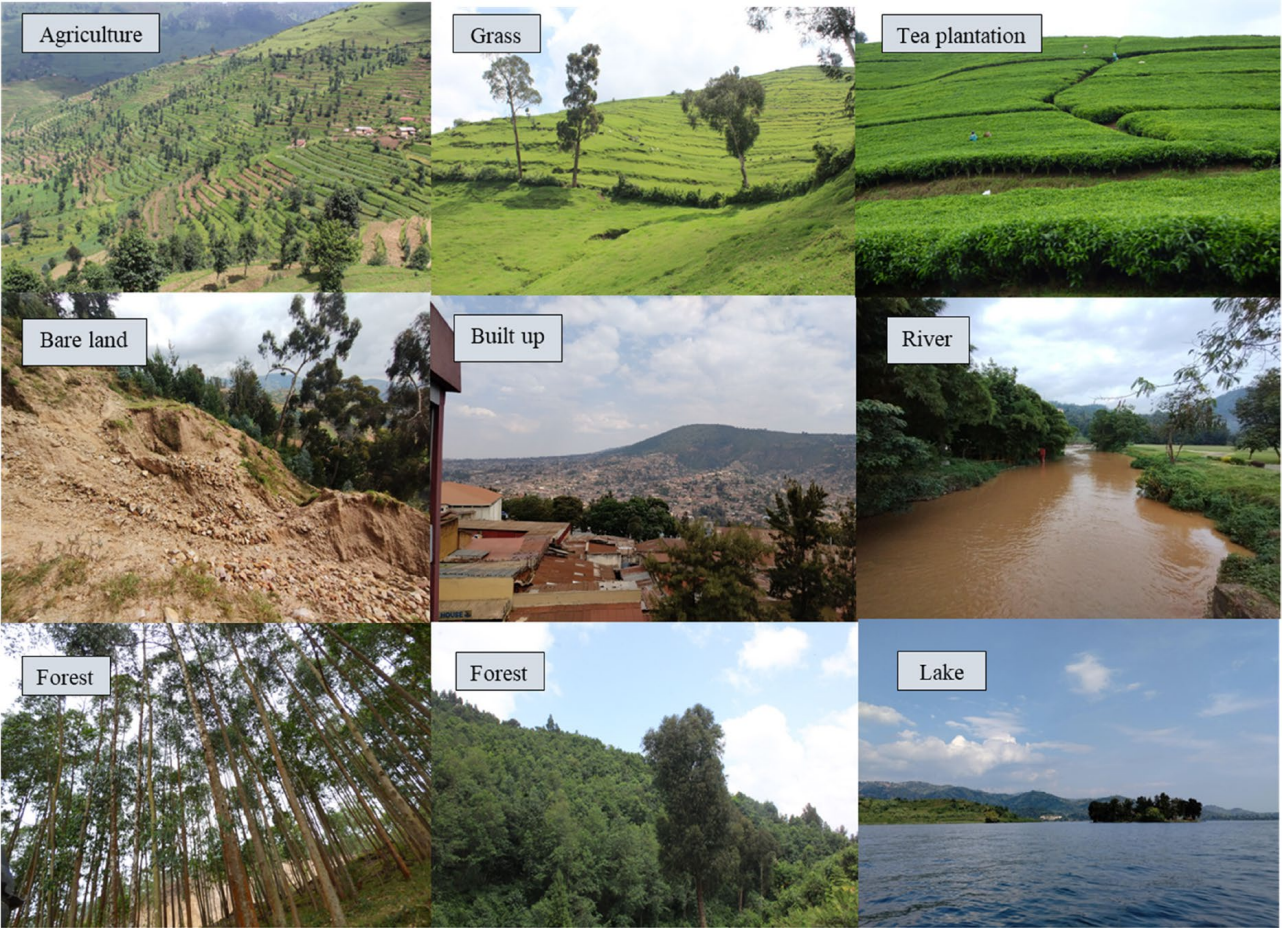
Pictures taken from the field showing examples of the 8 LULC classes considered

For the training process of supervised classification, reference points were directly gathered within the Google Earth Engine platform. This involved strategically making point markers across all eight classes (Fig. 4). The process was supplemented by historical Landsat imagery and Airbus archived images in Google Earth Pro for previous periods. About 1500 training points were gathered each year to ensure a robust training dataset.

The Landsat image collection was filtered over a 5-year span for each study period (as specified in Fig. 3), in alignment with Rwanda’s climatic seasons. The dry season was defined as June to August, while September to May was considered as the rainy season. A short dry season in December and January, often with occasional rainfall, was not included as part of the dry season. To represent each season, the composition method was employed to aggregate multiple Landsat images into a single image that represents the entire collection (Nasiri et al., 2022). This aggregation process enhances image quality by addressing cloud and shadow effects, filling data gaps, and compensating for irregular observations in Landsat images (Adepoju & Adelabu, 2020). The median reducer, chosen for its accuracy in synthetizing pixel values from multiple images, was used as the composite method. Images from both the dry and rainy seasons were aggregated separately before merging them to create seasonal composites that represent the characteristics of both seasons. This process helped to capture the unique features of each season, such as differentiating bare land from agriculture in dry season or delineating rivers during the rainy season. By merging images from both seasons, a wider range of spectral information is captured, enhancing feature discrimination and extraction of different LULC classes (Xu, 2007).

#### Classification and validation

The random forest machine learning algorithm, an ensemble model consisting of multiple decision trees, was trained using pre-defined training samples. The trained classifier was then applied to perform the classification of the entire image. This model was chosen for its effectiveness in managing the complex variability of the study area, enhancing prediction accuracy, and reducing the likelihood of overfitting (Mellor et al., 2013; Sibanda & Ahmed, 2021; Talukdar et al., 2020).

Evaluating the accuracy of remote sensing results is a crucial step. To this end, ground truth data provides reliable on-site information for validating the results. Consequently, an extensive field survey was undertaken from June to July 2023, to collect Ground Control Points (GCPs) within the LKV catchment. GPS coordinates were systematically recorded across all eight LULC classes (Fig. 4). The collected dataset first underwent a cleaning process to identify the most representative class per pixel, remove neighbouring GCPs that did not match the same LULC class, and eliminate duplicated GCPs to avoid over-representation. Additionally, quality control was conducted on the ground control points to ensure only those with stable LULC were considered, such as areas with older trees, while excluding points with recent changes. To create validation datasets for historical periods, the same approach as for training was used. This involved cross-referencing the collected data with archived Airbus images in Google Earth Pro and consulting local communities. Gathering historical evidence through discussions with local people provided valuable insights. Only data with high confidence were retained, resulting in a small dataset over time.

Table 1 presents the collected dataset, the cleaned validation dataset for 2020, and the retroactively validated datasets for 2010, 2000, and 1990. These GCPs were then used to generate an error matrix and calculate user, producer, and overall accuracies, as well as Kappa coefficients.

**Table 1:** Validation data per LULC classes

LULC/Years	Collected GCPs	Validation dataset			
		2020	2010	2000	1990
Agriculture	1523	463	225	166	129
Bare land	101	59	22	23	25
Built-up	489	146	93	52	43
Grass	351	150	77	66	72
Forest	985	347	136	68	65
River	130	67	43	17	17
Tea plantation	316	79	66	41	39
Lake	54	54	25	25	25
Total	2426	1,365	687	458	415

The classified images were then exported to GIS software for post-processing, where sieve filters were applied to remove noise and isolated misclassified pixels. Subsequently, statistical analysis was conducted to identify patterns and trends in LULC over time, and thematic maps were generated for further analysis.

### Change detection and prediction of future LULC scenarios

This study utilized the LCM-TerrSet, an integrated geospatial monitoring and modelling system for change detection and prediction of future LULC predictions. The Change Analysis tool within LCM was used to evaluate statistics and generate maps illustrating transitions that have occurred in the landscape over two successive time periods. Additionally, the Change Prediction tool was used to predict future LULC scenarios, incorporating statistical functions and algorithms that account for explanatory variables to understand their relationship with the observed class-transitions. The model considers historical images and incorporates prior knowledge of the study area to predict the future scenario based on change demands. This modelling approach has been widely applied, particularly in the context of land and water management (Bakr et al., 2022; Chuenchum et al., 2020; Gibson et al., 2018), wildfires management (Amato et al., 2018), and biodiversity and carbon emissions (Leta et al., 2021; Sangermano et al., 2012).

#### Explanatory variables for LULC changes

**Fig. 5:**
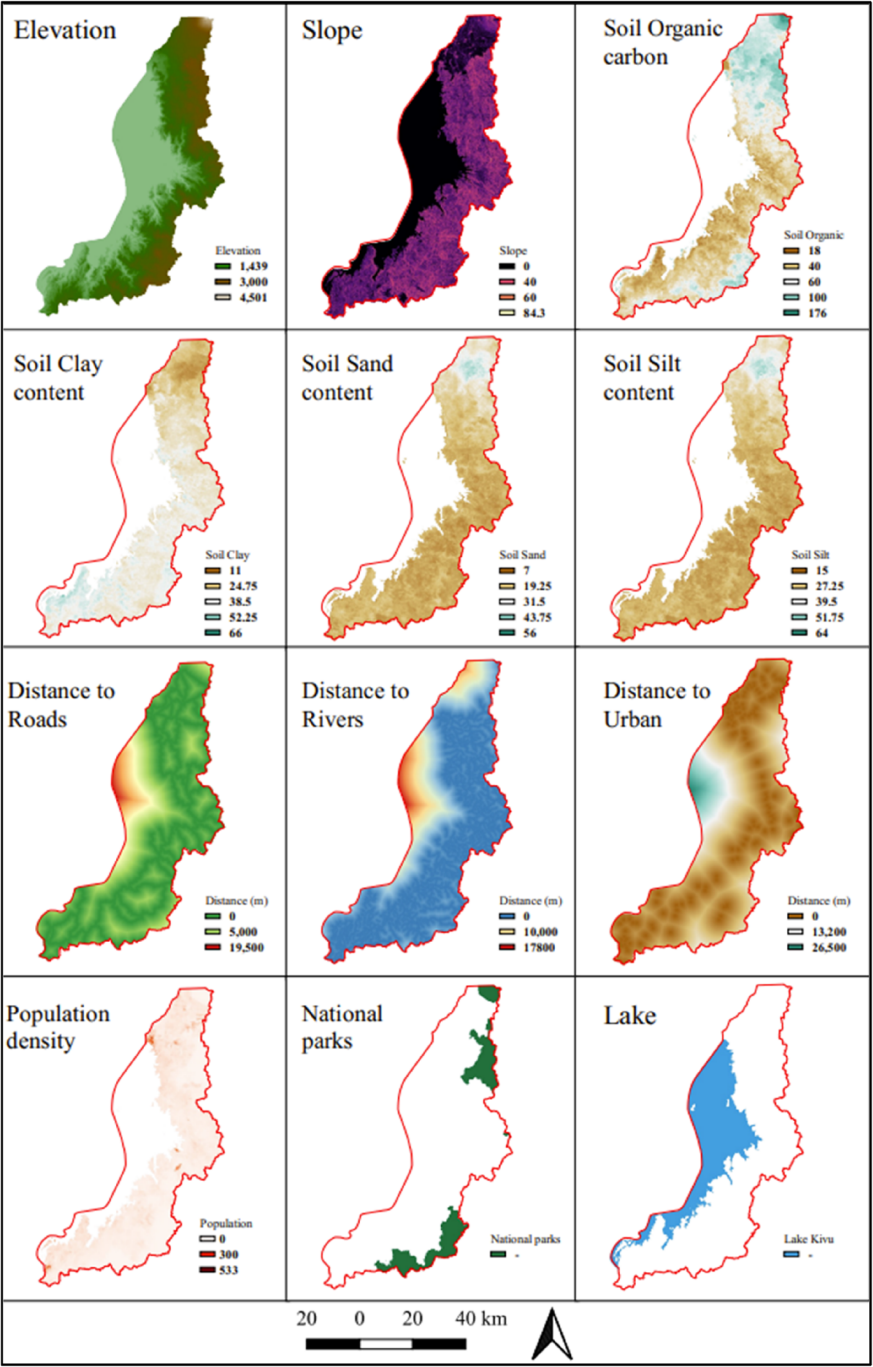
LULCC potential explanatory variables

Explanatory variables represent potential drivers responsible for observed changes in LULC. Their selection is crucial for ensuring the predictive performance of the model, as the forces that influenced past changes can be expected to influence future changes (Leta et al., 2021). In this study, explanatory variables encompassed diverse variables. Natural variables consisted of elevation, slope, soil texture (clay, sand, and silt), and soil organic carbon. Additionally, proximity-based variables, such as distance to roads, river networks, and urban centers, were incorporated. Urban centers were derived from the major urban areas using the classified 2020 Landsat image. Moreover, demographic variables such as population density, and specific land cover drivers, namely national parks and Lake Kivu, were also considered. Figure 5 shows graphically all the potential explanatory variables used in this study.

#### Model calibration and validation

LULC predictive modelling utilizes the spatially explicit variables that have driven past changes for model calibration. The calibration process involves systematically adjusting model parameters to improve the agreement between the predicted and observed transitions (Mandal et al., 2023). Multi-Layer Perception (MLP) neural network, recognized for its ability to simulate non-linear relationships and interactions within complex datasets (Gaur et al., 2020; Talukdar et al., 2020), was utilized to identify the rules governing the likelihood of transitions between LULC classes, referred to as transition potentials (Megahed et al., 2015). The MLP uses back propagation, iteratively adjusting variable importance by keeping each variable constant to assess its impact on the model’s predictive skill. This process is documented in the Backwards stepwise constant forcing report (Girma et al., 2022).

To accurately calibrate and validate the model, three maps at different time periods (*T*_1_, *T*_2,_ *T*_3_) are required. The study incorporated classified LULC maps from 2000 and 2010, along with potential explanatory variables, to establish an empirical change model. By integrating the MLP neural network, the model captured the transition potentials and predicted the 2020 LULC map using transition probability matrix. These probability maps provide estimates of the likelihood that each pixel will either convert to another class or persist (Hasan et al., 2020). The validation process plays an important role in gauging the model’s reliability and its effectiveness in predicting future LULC trends. To validate the model, the output predicted 2020 map was compared with the 2020 classified map. The LCM provides a built-in validation tool that generates a cross-tabulation map with three scenarios: “hits” areas where the model accurately predicted changes; “false alarms” areas where the model predicted changes that did not occur; and “misses” where the model failed to predict changes. This validation procedure was iteratively repeated until achieving a satisfactory agreement between the predicted and reference LULC map.

#### Prediction of future LULC scenarios

The calibrated hyper-parameters for MLP, determined through the validation process, were subsequently employed to predict future LULC scenarios. In LCM, transitions between two LULC maps are organized into transition sub-models, each representing a specific class transition. Transitions sharing similar underlying explanatory variables can be grouped into the same sub-model. Each sub-model is executed separately with its explanatory variables to generate transition potentials. In this study, transitions with a total area less than 300 ha were deemed negligible and excluded to avoid noise in the predictions. Initially, similar transitions (for instance, various sub-models leading to “Forest expansion”) were consolidated into a single sub-model. However, this approach often reduced the model’s accuracy and the skill measures. Consequently, each transition sub-model was evaluated individually.

The Markov Chain is implemented into LCM, to estimate the expected change rates for each pair of LULC classes. Subsequently, the change demand is applied, incorporating transition potentials, and considering planning strategies to allocate changes in the predicted LULC map. This process resulted in future LULC scenarios at *T*_4_, based on the LULC at *T*_3_ and the transition probability matrix from *T*_2_ to *T*_3_ (*P*_*ij*_). The probability is calculated based on Bayes theory that describes the probability of an event in the future based on prior knowledge (Mandal et al., 2023), as shown in the following equations:

$${T}_{4}={P}_{i,j}*{T}_{3}$$

$${P}_{i,j} = \left(\begin{array}{c}{P}_{\text{1,1}} {P}_{\text{1,2}}\dots {P}_{1,n}\\ {P}_{\text{2,1}} {P}_{\text{2,2}}\dots {P}_{2,n}\\ \dots \dots \dots \dots \\ {P}_{n,1} {P}_{n,2}\dots {P}_{n,n}\end{array}\right)\;\left(0\le {P}_{i,j}\le 1\right)$$

The model generates two predictions, the soft prediction indicating vulnerability scores ranging from 0 to 1; the hard prediction assigning each pixel to a specific LULC category based on a given threshold (Hasan et al., 2020; Sangermano et al., 2012). Table 2 presents the three LULC development scenarios predicted in this study, outlining their guiding principles, and associated policy measures or limitations influencing land transitions, referred to as incentives and constraints in LCM context. The scenarios include (1) Green Growth Economy (GGE), representing a continuation of ongoing LULCC patterns without considering negative impacts or mitigation measures; (2) Development of Anthropogenic Activities (DDA), focusing on urban and agricultural expansion to address population growth: and (3) Enhanced Forest Protection (EFP), prioritizing forest conservation and expansion.

**Table 2:** Future LULC SCENARIOS development

LULC scenarios	Description	Incentives/Constraints
Green Growth Economy (GGE)	This scenario is in line with the ongoing land use trend, aiming for the sustainable land use practices	It is driven solely by 2010–2020 land transitions without imposing any constraints or incentives
Development of Anthropogenic Activities (DAA)	This scenario considers ongoing human activities, with a focus on expanding urban and agricultural land to cope with population growth	It is consistent with land transition trends for 2010–2020, maintaining the existing urban and agricultural areas. Transitions from Built-up and Agriculture to other classes are excluded in the end-point generation
Enhanced Forest Protection (EFP)	This scenario prioritizes measures to increase forested areas, aiming to protect and enhance forest growth	It emphasizes preserving existing forests, excluding transitions from forest to other classes in endpoint generation. Additionally, it imposes constraint on National Parks to prevent any further land conversion

## Results

### Accuracy assessment

To validate the classification results, independently gathered ground truthing data from field surveys were used to compute an error matrix. This facilitated the evaluation of user (UA), producer (PA), and overall accuracies. Additionally, the Kappa index was computed to assess the classifier performance. The resulting accuracy for the respective study periods and LULC classes is presented in Table 3.

**Table 3:** Accuracy assessment

LULC/Classes accuracy	1990		2000		2010		2020	
	UA (%)	PA (%)	UA (%)	PA (%)	UA (%)	PA (%)	UA (%)	PA (%)
Agriculture	81	87	87	82	84	89	88	81
Bare land	72	90	83	83	82	90	78	75
Built-up	98	93	94	89	96	97	75	89
Grass	93	84	86	92	99	88	81	95
Forest	86	79	91	86	98	90	89	88
River	82	93	53	100	63	93	88	100
Tea plantation	95	95	80	94	100	87	95	86
Lake	100	100	100	100	100	100	100	100
Overall accuracy	87		87		91		87	
Kappa	0.83		0.84		0.88		0.83	

As expected, the Lake class showed the best accuracy, with 100% for both UA and PA across all study periods. In contrast, River class showed the lowest accuracy, particularly under UA, with 53% and 63% in 2000 and 2010, respectively. This low accuracy can be attributed to the turbidity of the river water in the region, which potentially leads to misrepresentation as bare land. Despite some inconsistency and fluctuations in the accuracies across different LULC classes and study periods, the overall accuracy and Kappa index are high, especially in 2010 with 91% and 88% respectively, demonstrating the reliability of the classification results.

### LULC classification

**Fig. 6:**
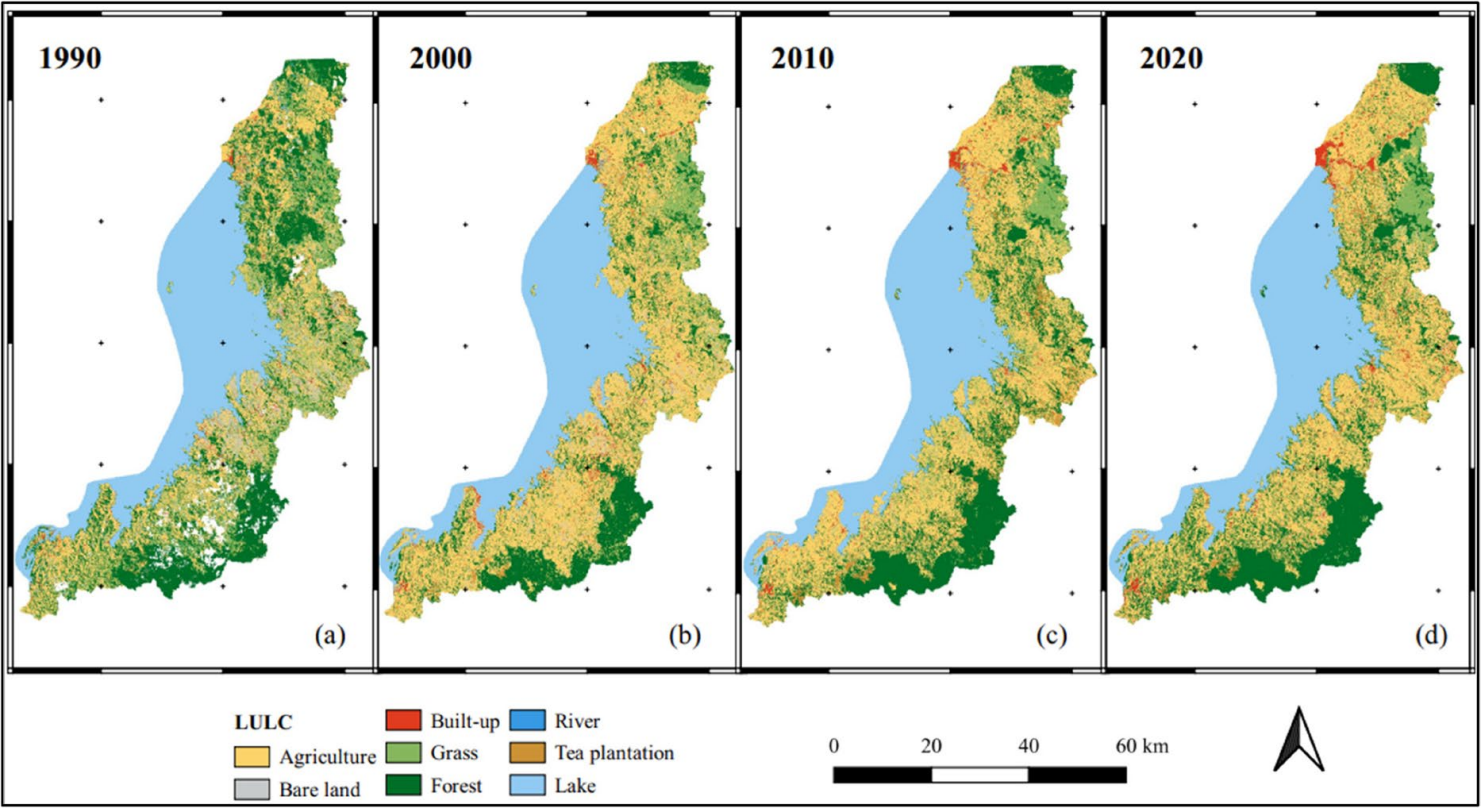
LULC maps for the four investigated periods: 1990, 2000, 2010, and 2020

**Fig. 7:**
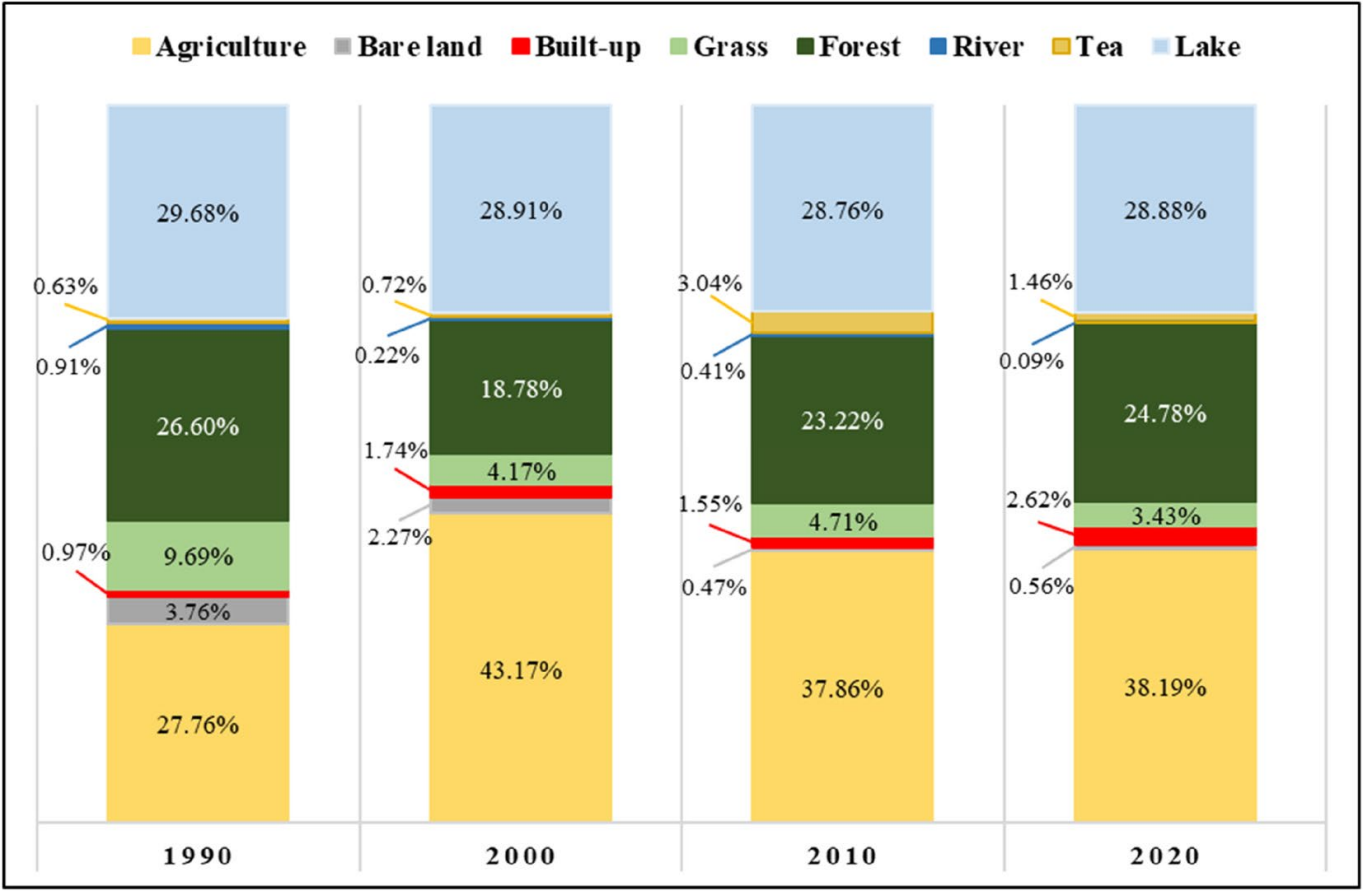
Comparative chart of the percentage of LULC classes across the study period

This section presents the results of the LULC classification. Figure 6 illustrates the LULC classified maps for the four consecutive periods investigated in this study. Concurrently, Fig. 7 provides a summary of statistics, depicting the percentage of each LULC class relative to the total study area.

During the first decade (1990–2000), the LKV catchment experienced a substantial increase in agriculture throughout the catchment, contributing to a significant loss of forest cover, particularly in the northern and some southern parts of the catchment. Statistical analysis indicates a significant increase in agricultural land, rising from 27.76% in 1990 to 43.17% in 2000 (a net increase of 565 km^2^). Concurrently, there was a considerable decrease in forest cover from 26.6 to 18.78% (a net decrease 244 km^2^), with similar trends in grassland and bare land. Additionally, built-up areas nearly doubled, expanding from 32 to 62 km^2^.

In the subsequent decade (2000–2010), there was a decline in agricultural land expansion, primarily in the central and southern parts, along with a slowdown in forest loss and some gains in the central catchment area. The forest trend exhibited a 5% increase (a net gain of 154 km^2^), whereas agriculture experienced a 6% decrease (a net decline of 184 km^2^), with concurrent gains in grassland and tea plantations. Similarly, the last decade (2010–2020) portrayed a continuation of ongoing forest regrowth and a dynamic balance between expansion and reduction of agricultural land. This period witnessed a notable growth in built-up areas, expanding by 37 km^2^, representing a 160% increase compared to the previous decade. Additionally, there was an increase in forest cover and agricultural land, by 54 km^2^ and 11 km^2^, respectively, along with a decrease in tea plantation and grassland by 54 km2 and 44 km^2^, respectively.

These reciprocal changes between forested and agricultural land underscore a dynamic interplay between these two LULC classes, emphasizing their important role in shaping the region’s landscape. Moreover, the built-up class exhibited a consistent increase throughout the study period. The corresponding change maps can be found in the supplementary materials.

### Future LULC predictions

#### LULC transition potentials and explanatory variables

**Fig. 8:**
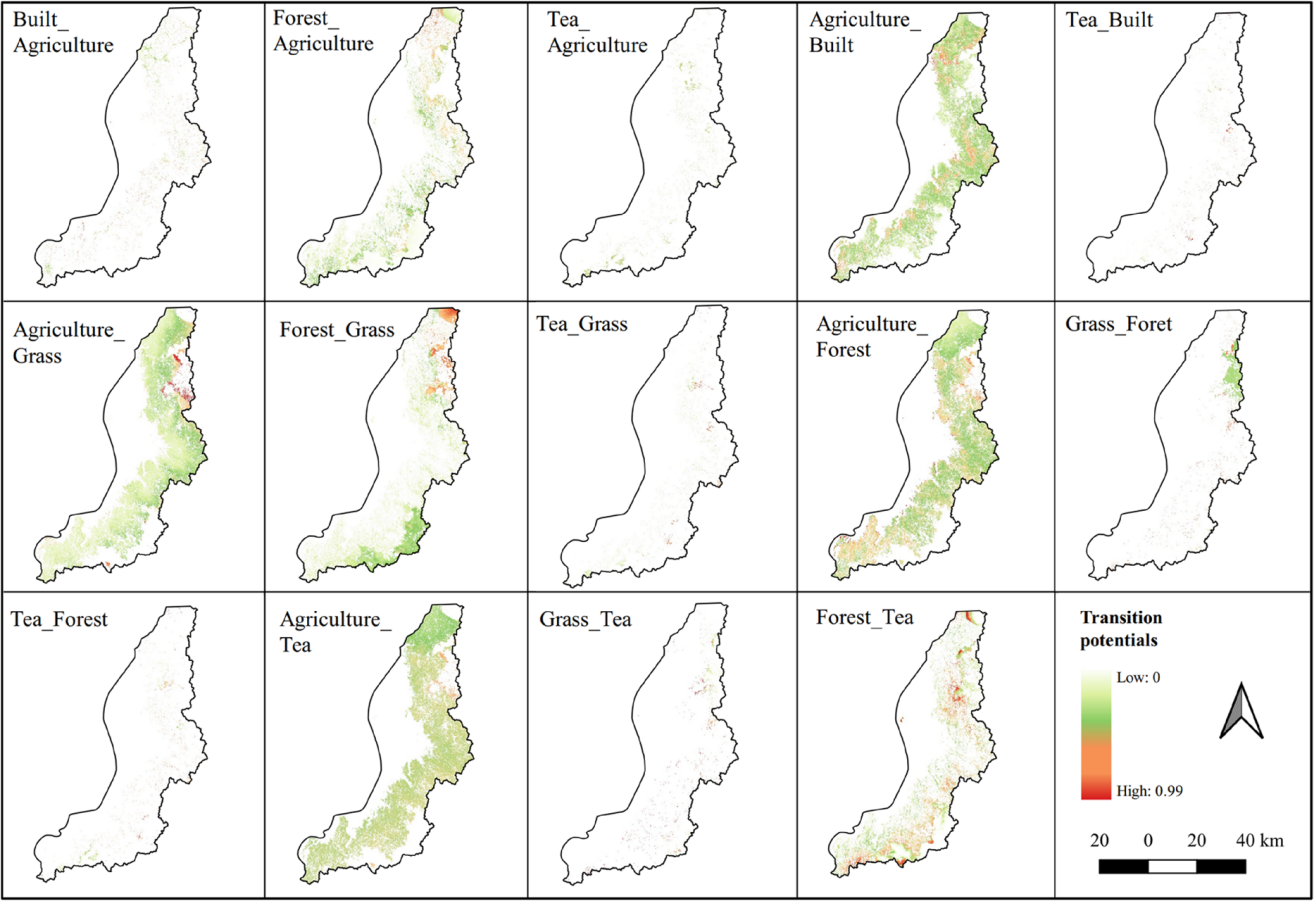
Representation of transition potentials derived from the 2010–2020 trend, and their respective probabilities

The LCM identified 18 distinct transition sub-models between 2010 and 2020, which were subsequently used to calculate the transition potentials. Consequently, the MLP neural network highlighted explanatory drivers responsible for each sub-model and their corresponding weights. Table 4 presents a detailed information on the 18 transition sub-models found in this study, with their respective accuracy and skill measures. It highlights also the top six explanatory variables responsible for each transition sub-model. Additionally, Fig. 8 spatially illustrates the transition potentials and associated probabilities (ranging from 0 to 0.99), providing insights into the likelihood of transitions between various LULC classes.

**Table 4:** LULC transition sub-models, accuracy and skill measures, and their driving variables

Transition sub-model	AE1 (Bare to Agriculture)	AE2 (Built to Agriculture)	AE3 (Grass to Agriculture)	AE4 (Forest to Agriculture)	AE5 (River to Agriculture)	AE6 (Tea to Agriculture)
Accuracy rate	71.08%	71.28%	73.63%	74.5%	71.88%	79%
Skill measure	0.42	0.62	0.47	0.5	0.44	0.6
Top 6 driving variables	*1. Population density*	*1. Population density*	*1. Population density*	*1. Slope*	1. Soil-organic	*1. Population density*
	2. Soil- organic	*2. Slope*	2. Soil-organic	2. Distance to rivers	2. Soil-clay content	2. Distance to rivers
	3. Distance to rivers	3. Parks	3. Distance to rivers	*3. Distance to urban*	3. Population density	*3. Slope*
	*4. Distance to urban*	*4. Distance to urban*	*4. Distance to urban*	*4. Population density*	4. Soil-Sand content	4. Soil-organic
	*5. Slope*	5. Distance to rivers	*5. Slope*	5. Soil- organic	*5. Slope*	*5. Distance to urban*
	6. Soil-clay content	6. Elevation	6. Parks	6. Soil- clay content	6. Distance to rivers	6. Soil-organic
Transition sub-model	UE1 (Agriculture to Built)	UE2 (Bare to Built)	UE3 (Tea to Built)	GE1 (Agriculture to Grass)	GE2 (Forest to Grass)	GE3 (Tea to Grass)
Accuracy rate	66.82%	67.74%	88.65%	83%	79.64%	83.25%
Skill measure	0.4	0.4	0.77	0.66	0.6	0.67
Top 6 driving variables	1. Distance to rivers	*1. Slope*	1. Distance to rivers	1. Distance to rivers	*1. Population density*	1. Soil-organic
	*2. Distance to urban*	2. Distance to rivers	*2. Distance to urban*	*2. Distance to urban*	2. Soil-organic	2. Distance to rivers
	*3. Population density*	*3. Distance to urban*	*3. Population density*	*3. Population density*	3. Distance to rivers	*3. Distance to urban*
	*4. Slope*	*4. Population density*	*4. Slope*	*4. Slope*	*4. Distance to urban*	*4. Population density*
	5. Soil-organic	5. Soil-organic	5. Soil-organic	5. Parks	*5. Slope*	*5. Slope*
	*6. Elevation*	6. Roads	*6. Elevation*	*6. Elevation*	*6. Elevation*	*6. Elevation*
Transition sub-model	FE1 (Agriculture to Forest)	FE2 (Grass to Forest)	FE3 (Tea to Forest)	TE1 (Agriculture to Tea)	TE2 (Grass to Tea)	TE3 (Forest to Tea)
Accuracy rate	61.7%	77.07%	77.64%	63.46%	93.37%	75.43%
Skill measure	0.5	0.54	0.55	0.27	0.87	0.51
Top 6 driving variables	*1. Population density*	*1. Population density*	*1. Population density*	*1. Population density*	1. Distance to rivers	1. Distance to rivers
	2. Distance to rivers	*2. Distance to urban*	*2. Slope*	*2. Slope*	*2. Distance to urban*	2. Parks
	*3. Slope*	3. Distance to rivers	*3. Distance to urban*	*3. Distance to urban*	*3. Population density*	*3. Distance to urban*
	*4. Distance to urban*	*4. Slope*	4. Distance to rivers	4. Distance to rivers	*4. Slope*	*4. Population density*
	5. Soil-organic	5. Parks	5. Soil-organic	5. Soil-organic	5. Elevation	*5. Slope*
	6. Soil-silt content	6. Soil- organic	6. Distance to roads	6. Parks	6. Soil-organic	6. Elevation

#### Future LULC scenarios

**Fig. 9:**
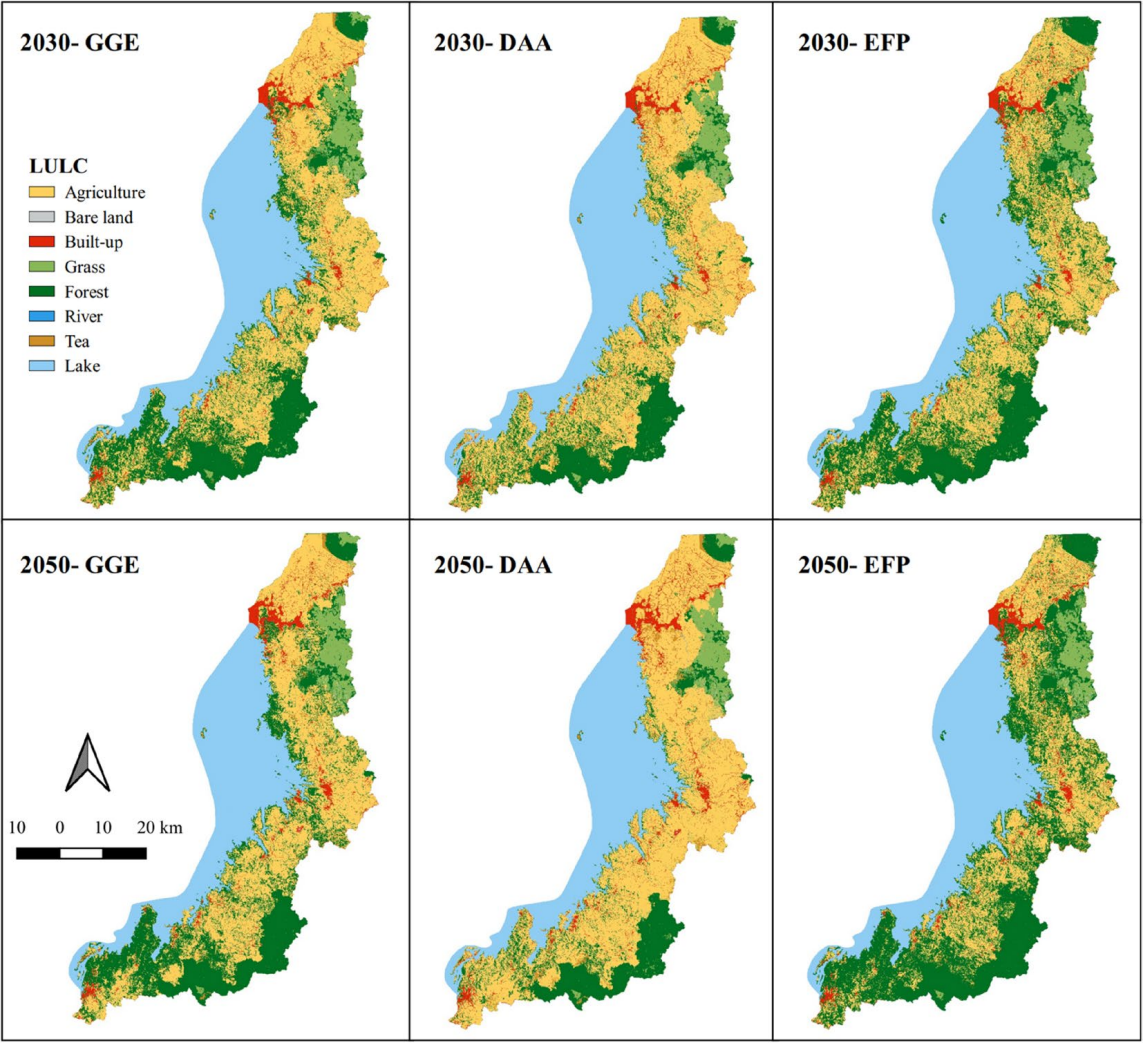
Predicted LULC scenarios for 2030 and 2050 under Green Growth Economy (GGE), Development of Anthropogenic Activities (DAA), and Enhanced Forest Protection (EFP)

**Fig. 10:**
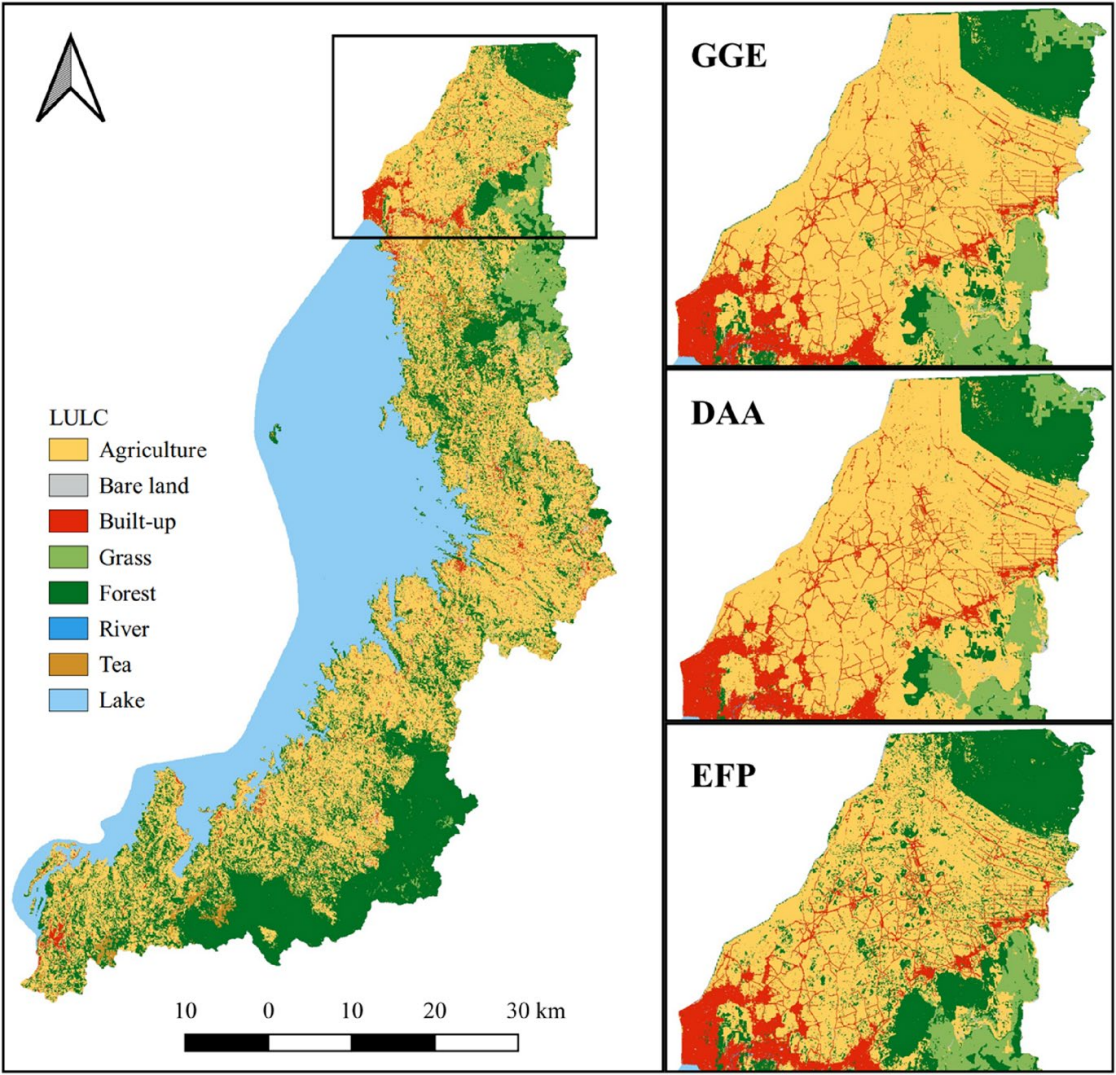
Exploring diverse LULC scenarios in a small heterogeneous area

Figure 9 illustrates the predicted LULC maps, representing three development scenarios for the years 2030 and 2050. Additionally, Fig. 10 offers a detailed view of a heterogenous region within the northern catchment, to illustrate the distinct LULC patterns across the three LULC scenarios. For a comprehensive overview, Table 5 provides statistical information corresponding to each scenario.

**Table 5:** Statistics on future LULC Scenarios for 2030 and 2050

LULC categories/ scenarios	2030						2050					
	GGE		DAA		EFP		GGE		DAA		EFP	
	km^2^	%	km^2^	%	km^2^	%	km^2^	%	km^2^	%	km^2^	%
Agriculture	127,725	36.82	143,369	41.33	111,066	32.02	123,720	35.67	155,412	44.8	90,702	26.15
Bare land	489	0.14	489	0.14	489	0.14	468	0.13	468	0.13	468	0.13
Built-up	11,281	3.25	14,502	4.18	11,281	3.25	12,466	3.59	17,589	5.07	12,466	3.59
Grass	14,661	4.23	16,177	4.66	13,492	3.89	15,413	4.44	18,637	5.37	13,161	3.79
Forest	88,594	25.54	67,531	19.47	107,024	30.85	91,134	26.27	49,711	14.33	127,384	36.72
River	135	0.04	135	0.04	135	0.04	163	0.05	163	0.05	163	0.05
Tea plantation	3,839	1.11	4,522	1.3	3,237	0.93	3,359	0.97	4,743	1.37	2,379	0.69
Lake	100,139	28.87	100,139	28.87	100,139	28.87	100,139	28.87	100,139	28.87	100,139	28.87

The future LULC scenarios for the periods of 2030 and 2050 will likely reveal significant variations in agricultural land, built-up and forest area. Minor alterations are expected in the tea plantation, river, and grass classes. The EFP scenario indicates a notable increase in forest cover compared to other scenarios. In contrast, the DAA scenario anticipates substantial growth in built-up and agricultural land. While the GGE scenario represents a moderate projection, maintaining the existing status quo without drastic shifts.

Agriculture is predicted to remain the largest LULC class in the catchment, with fluctuations depending on the scenario. The EFP is expected to result in a significant reduction in agricultural areas, by almost 10% compared to DAA for both 2030 and 2050. Additionally, human activities are expected to have a profound impact on forested area, leading to a substantial difference in forest cover predictions between DAA and EFP scenarios. For 2030, the forest cover is predicted to be 19% under DAA compared to 30% under EFP, and for 2050, it is expected to be 14% under DAA versus 37% under EFP.

## Discussion

### Historical LULCC

Over the last three decades, the LKV catchment has witnessed considerable LULCC, driven by political, socio-economic, and demographic variables. Notably, the initial decade considered in this study (1990–2000) revealed the most substantial changes, characterized by a significant forest loss, and marked rise in urban and agricultural land. These changes are partly attributed to the early 1990s conflicts in the region, when forests became battle zones due to their strategic locations (Arakwiye et al., 2021; Plumptre, 2003). Additionally, the region witnessed major population movements in the aftermath of the 1994 genocide against Tutsis in Rwanda. This includes the return to Rwanda of people who had fled during the war and genocide periods from 1959 as wells as the influx of refugees due to the civil wars in the Democratic Republic of Congo in the late 1990s and early 2000s (Bagalwa et al., 2016; Mugiraneza et al., 2019). These pressures led to an extensive conversion of forested land for settlement and agricultural expansion, to meet the growing demands for food and energy (Munanura et al., 2018; Rukundo et al., 2018). This period was defined by the fragmentation of natural forest parks, with Gishwati-Mukura in the LKV catchment losing more than half of its original land during the period (Kanyamibwa, 1998; Muhire et al., 2021).

In the second (2000–2010) and third (2010–2020) decades considered in this study, the region experienced contrasting trends in LULCC. The findings revealed a regrowth of forest cover and an equilibrium between expansion and reduction of agricultural land. This shift mirrors Rwanda’s strategic initiatives aiming at fostering sustainable environmental and natural resource management, transitioning towards a green economy. This commitment is reflected in the country’s Green Growth Strategy, focusing on addressing environmental challenges and land degradation. The increase in forest cover aligns with several of the country’s policies and strategic goals. These include the increasing forest cover to 30% by 2020 (Akinyemi, 2017) and developing the Rwanda National Land Use Development Master Plan (NLUDMP), which further optimized the land use across the landscape (Banerjee et al., 2020). Rwanda’s Environment and Natural Resources (ENR) sector proposes rational use of natural resources to optimize future land use management as per the country’s NLUDMP, anchored in a detailed understanding of LULC at high resolution. Results of the present study also indicate the expansion of larger forest and grassland patches, particularly in the third decade, with occasional forest fragmentation amid agricultural land. This can be interpreted as a consequence of the country’s policy on land consolidation (Bizoza, 2021), following the initiation of soil and water conservation measures such as agroforestry and terracing, to sustain the agricultural land.

Furthermore, results showed a continuous increase in built-up areas, with a peak of about 160% in the last decade compared to the second decade. The LULC maps along time highlight this urban growth, particularly in the North and Southern parts of the catchment. This trend coincides with the observed urban sprawl. Factors contributing to this expansion include rapid population growth, infrastructure development, and overall urbanization processes in these areas (Amisi et al., 2022). Additionally, there is a consistent decrease in the area covered by the river, which can be attributed to measures taken for riverbank protection such as the planting of trees. It is important to note, however, that the results may not entirely reflect the reality, given that rivers within the LKV catchment are too narrow to be accurately detected using 30 m satellite image resolution.

The results of LULCC patterns in LKV align with the global trends observed in tropical catchments, where widespread deforestation and the expansion of anthropogenic activities were predominant in the decades leading up to the 2000s. However, a noticeable reverse trend have been observed in the post-2000, with afforestation and reforestation efforts gaining momentum (Kayitesi et al., 2022). This change in direction is largely due to the implementation of various global and regional initiatives aimed at forest restoration and the promotion of sustainable land use practices including New York Declaration on Forests, Bonn Challenge, and African Forest Landscape Restoration Initiative (Dave et al., 2018).

### Explanatory variables and future LULC scenarios

The analysis of explanatory variables for LULCC revealed that factors including proximity to urban centres and population density play a significant role in most LULC transitions. This aligns with other studies, mainly in developing countries (Bongasie et al., 2024; Khwarahm et al., 2021; Mariye et al., 2024), highlighting the role of urbanization and population growth in influencing LULC dynamics. Terrain slope emerges as another key factor, especially in transitions between agriculture, grassland, and forest categories, confirming the significant role of the physical landscape in determining LULCC patterns, as supported by other studies (Akintuyi et al., 2021; Mandal et al., 2023).

In terms of agricultural land expansion, proximity to rivers stands as one of the primary drivers, underscoring the dependence on river water for irrigation, and the subsequent growth in agricultural areas along these water sources. This factor was also found to be influential for grassland expansion, associated primarily with pastureland, underlying the significance of water access for grazing and livestock needs, in agreement with findings from various studies (Najmuddin et al., 2018; Wassenaar et al., 2007). As revealed by (Li et al., 2021) soil organic content was specifically distinguished as a significant factor for agricultural land extension, while soil texture categories played a role for other LULC classes, though with less impact. While the presence of parks did not show a significant impact on forest expansion, implying that factors other than their presence may shape forest changes, it is important to highlight that these protected areas effectively discourage encroachment by nearby communities (Riggio et al., 2019).

The predicted future LULCC based on three scenarios indicates distinct trajectories in the LKV catchment for both 2030 and 2050. The GGE scenario envisions a continuation of the existing land use trends in line with ongoing forest conservation efforts. It highlights evidence of positive environmental outcomes, notably in the promotion and protection of forests. This suggests that the existing policy framework in Rwanda is already effective in influencing LULC outcomes favourably (Bullock et al., 2021), which may not be as pronounced in other developing countries, as showcased by various case studies (Dietz et al., 2023; Khwarahm et al., 2021; Mungai et al., 2022). The EFP scenario further enhances these outcomes, aiming for increased environmental sustainability by preserving existing forested land and actively fostering forest expansion on other lands, alongside the protection of national parks. However, this scenario may pose a challenge to agriculture, which is crucial for food security (Bullock et al., 2021). Conversely, the DAA scenario presents the opposite situation, emphasizing the growth of urban and agricultural lands, potentially conflicting with ongoing forest conservation. Therefore, the GGE scenario emerges as an intermediate option, advocating for a balance between economic progress and environmental conservation.

Future LULC scenarios are anticipated to interact significantly with various environmental hazards. Rwanda has experienced a 2.3% annual population growth rate between 2012 and 2022. According to the National Institute of Statistics Rwanda, the population is projected to reach 16.4 million by 2032 and 23.6 million by 2052 (NICR, 2023). Accommodating this increase in population will necessitate more space for settlement and agricultural land, potentially leading to the predicted DAA scenario. Such expansion could intensify environmental hazards, including floods, landslides, and soil erosion (Avashia & Garg, 2020; Remondi et al., 2016). Therefore, strategic measures are needed to mitigate these effects. Concurrently, research has demonstrated the effectiveness of green strategies like GGE and EFP in managing environmental hazards like floods and landslides (Locatelli et al., 2020; Nickel et al., 2014). However, these scenarios emphasizing sustainable development and conservation measures, could serve as counterbalance to the pressures of demographic expansion and urbanization. Moreover, the direction of future LULC will depend on a variety of different factors, including demographic trends, socio-economic developments, and the ongoing climate change.

### Methodological considerations

This study employed an integrated approach, combining the capabilities of Google Earth Engine for LULC classification, coupled with Land Change Modeler for change detection and prediction of future LULC scenarios in LKV catchment. The methodology involved the use of merged seasonal composites to capture the dynamic seasonal variations of the landscape. This, combined with the use of spectral indices such as NDVI, NDWI, and NDBI, along with topographic features like slope and elevation, significantly improved the discrimination and extraction of different LULC classes (Onyango & Opiyo, 2022; Triscowati et al., 2019). This was particularly effective in the heterogenous and seasonally variable landscape of the LKV catchment. Nevertheless, getting a sufficient number of satellite images, was a challenge, particularly in the 1990s and 2000s where the few available images were mostly clouded. To address this, Landsat images with a 5-year span have been aggregated to represent each study period using a median composite. This approach may compromise the capture of short-term LULC dynamics, reflecting a trade-off between image availability and the ability to capture temporal changes over shorter periods.

Future LULC scenarios were predicted based on the influence of natural and socio-economic explanatory variables on historical LULC class transitions. The Multi-Layer Perceptron neural network allowed the model to efficiently estimate the potential for various land use transitions, enabling predictions of future land use patterns. However, the LKV catchment faces various uncertainties in terms of population change and increased demand for land. Nevertheless, this study presents a methodology for quantifying future LULC scenarios with different development pathways (Bakr et al., 2022; Shafie et al., 2023). Thereby providing a comprehensive assessment of potential outcomes under different conditions. One potential way for enhancing the future prediction of LULC scenarios is to incorporate the Rwanda master plan for 2050, a document that was not accessible during the research phase. This master plan is a crucial policy document intended to guide the LULC development and could significantly shape future landscape transformations. The integration of this plan into future LULC scenario modelling would substantially enhance the accuracy and applicability of future LULC scenarios.

## Conclusion and recommendations

This study conducted image classification in Google Earth Engine using random forest classifier by incorporating merged seasonal composites Landsat images acquired across dry and rainy seasons, topographic features, and three spectral indices (NDVI, NDWI, and NDBI). This approach significantly enhanced the feature discrimination and extraction of different LULC classes, making it particularly effective for detailed and accurate LULC analysis. In addition, by integrating MLP neural network from LCM toolset, this study simulated potential future LULC scenarios based on classified images coupled with demographic, natural, and socio-economic explanatory variables.

To the best of our knowledge, this is the first study on LKV catchment that has applied an advanced geo-processing approach coupling remote sensing and machine learning for LULC assessment and prediction. This approach has been utilized both for image classification and the development of future scenarios. In a broader context, these modern methodological improvements need to be more extensively applied in Earth studies, especially in developing countries in the Global South.

The comprehensive analysis of LULC dynamics within the LKV catchment revealed significant changes over the past three decades. Despite forest cover losses in the 1990s, a post-conflict period, there has been a remarkable forest regrowth since the 2000s, largely attributed to Rwanda’s drive towards a green economy. The study recommends adopting the Green Growth Economy scenario aligned with ongoing conservation measures. It advocates for sustainable land use and change management to balance conservation goals with the needs of Rwanda’s increasing population.

This research highlights existing environmental challenges in the catchment due to LULCC, emphasizing the need for more in-depth studies to understand the impacts of LULCC on environmental hazards in LKV catchment. The study also recommends further research to include analysis of future LULC scenarios, especially considering population growth and migration trends, along with the integration of 2050 national development master plans, to enhance LULC projections.

The findings of this study can potentially support the planning and implementation of sustainable natural resource management, aimed at preserving the ecological integrity of the lake and its surrounding landscapes. Future LULC scenarios provide insights into the potential environmental and socio-economic implications of different development pathways. Finally, this research contributes to identifying opportunities for land restoration, and conservation efforts in LKV catchment, in alignment with national goals including Rwanda’s Green Growth Strategy, Nationally Determined Contributions (NDC), among others, and global goals, including Bonn Challenge, Sustainable Development Goals, and Kunming-Montreal Global Biodiversity Framework (GBF) targets by enhancing land management, biodiversity conservation, and climate resilience.

## Supplementary Information

Below is the link to the electronic supplementary material.

Supplementary file1 (DOCX 14 KB)

Supplementary file2 (DOCX 733 KB)

## Data Availability

Data will be made available on request.
