# Supplementary material for: Land use land cover change in the African Great Lakes Region: a spatial–temporal analysis and future predictions
Source: Environ Monit Assess. 2024 Aug 27;196(9):852. doi: 10.1007/s10661-024-12986-4 (PMC11349865; doi:10.1007/s10661-024-12986-4)
Supplement: Supplementary file 1 — Supplementary file1 (DOCX 14 KB) [file 10661_2024_12986_MOESM1_ESM.docx]

**Google Earth Engine script for image classification**

Script link: <https://code.earthengine.google.com/?scriptPath=users%2Fkanaomie%2FCongo_Basin%3ALULC_2020_Script>

Links for assets:

- Catchment boundary: <https://code.earthengine.google.com/?asset=users/kanaomie/Sebeya/Lake_Kivu_catchment>
- Validation data set: <https://code.earthengine.google.com/?asset=users/kanaomie/Sebeya/MergedGT_20>
