# Supplementary material for: Land use land cover change in the African Great Lakes Region: a spatial–temporal analysis and future predictions
Source: Environ Monit Assess. 2024 Aug 27;196(9):852. doi: 10.1007/s10661-024-12986-4 (PMC11349865; doi:10.1007/s10661-024-12986-4)
Supplement: Supplementary file 2 — Supplementary file2 (DOCX 733 KB) [file 10661_2024_12986_MOESM2_ESM.docx]

**Supplementary materials.**

Table 1: LULC change matrix for the years 1990, 2000, 2010 and 2020, in the LKV catchment, Rwanda.

| **LULC/**  **Area** | **1990** | | **2000** | | **2010** | | **2020** | |
| --- | --- | --- | --- | --- | --- | --- | --- | --- |
|  | **km^2^** | **%** | **km^2^** | **%** | **km^2^** | **%** | **km^2^** | **%** |
| Agriculture | 935.65 | 27.76 | 1500.20 | 43.17 | 1316.52 | 37.86 | 1327.73 | 38.19 |
| Bare land | 126.83 | 3.76 | 79.02 | 2.27 | 16.22 | 0.47 | 19.32 | 0.56 |
| Built-up | 32.73 | 0.97 | 60.55 | 1.74 | 53.77 | 1.55 | 91.08 | 2.62 |
| Grass | 326.53 | 9.69 | 144.94 | 4.17 | 163.64 | 4.71 | 119.35 | 3.43 |
| Forest | 896.60 | 26.60 | 652.73 | 18.78 | 807.26 | 23.22 | 861.69 | 24.78 |
| River | 30.61 | 0.91 | 7.72 | 0.22 | 14.09 | 0.41 | 3.09 | 0.09 |
| Tea plantation | 21.09 | 0.63 | 25.10 | 0.72 | 105.63 | 3.04 | 50.68 | 1.46 |
| Lake | 1000.35 | 29.68 | 1004.64 | 28.91 | 1000.01 | 28.76 | 1004.07 | 28.88 |


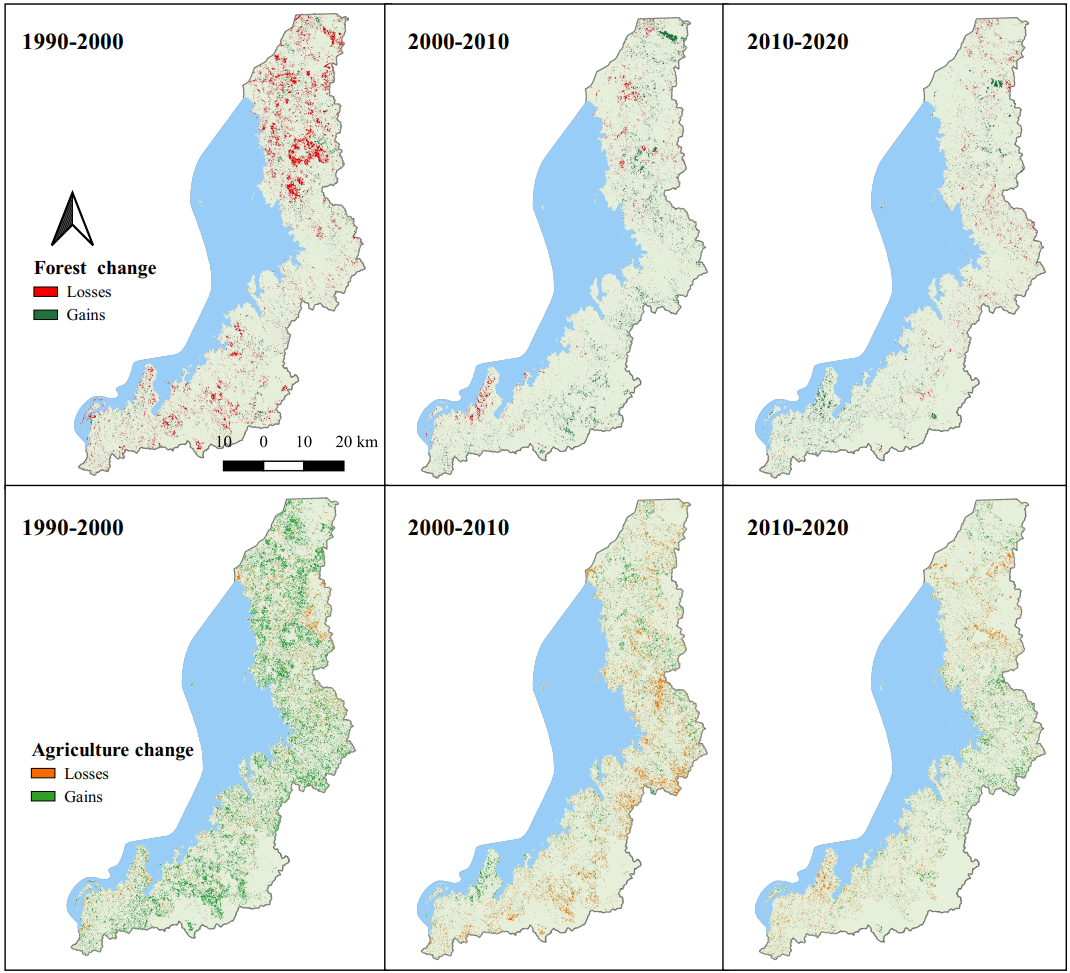


Figure 1: Forest and agriculture losses and gains for (a) first decade, (b) second decade, and (c) third decade.
